# Clinical Outcomes of Stereotactic Body Radiotherapy (SBRT) for Oligometastatic Patients with Lymph Node Metastases from Gynecological Cancers

**DOI:** 10.3390/jpm13020229

**Published:** 2023-01-27

**Authors:** Giuseppe Facondo, Gianluca Vullo, Vitaliana De Sanctis, Margherita Rotondi, Riccardo Carlo Sigillo, Maurizio Valeriani, Mattia Falchetto Osti

**Affiliations:** Department of Medicine and Surgery and Translational Medicine, Sapienza University of Rome, Radiotherapy Oncology, St. Andrea Hospital, 00189 Rome, Italy

**Keywords:** stereotactic body radiotherapy, oligometastases, lymph nodes, gynecological cancer

## Abstract

Background: To evaluate clinical outcomes of stereotactic body radiation therapy (SBRT) as a local treatment for lymph node metastases from gynecological cancers. Methods: Between November 2007 and October 2021, we retrospectively analyzed 29 lymph node metastases in 22 oligometastatic/oligoprogressive patients treated with SBRT. The Kaplan–Meier method was used to estimate the rates survival. Univariate analysis for prognostic factors were performed with the log-rank test, and Cox proportional hazards regression was used to estimate hazard ratios (HR). Results: Median age was 62 years (IQR, 50–80 years). Median follow-up was 17 months (IQR 10.5–31 months). The median survival was 22 months (CI 95%: 4.2–39.7, IQR: 12.5–34.5 months). Six months, one year and two year overall survival (OS) were 96.6%, 85.2%, and 48.7%, respectively. Median local control (LC) was not reached. Six months, 1one year and 2 year were 93.1%, 87.9%, and 79.9%, respectively. Distant metastasis free survival (DMFS) at one year, and two year was 53% and 37.1%, respectively Four patients (18%) experienced acute G1–G2 toxicities. No G3–4 acute toxicity was reported, and no late toxicity was observed. Conclusions: SBRT for lymph node recurrence offers excellent in-field tumor control with safe profile and low toxicities. Size, number of oligometastases, and time primary tumor to RT seem to be significant prognostic factors.

## 1. Introduction

In the last decade, stereotactic body radiotherapy (SBRT) has gained considerable relevance as a treatment option in different settings and stages of gynecological malignancies [1,2]. More recently, the increasing use of imaging exams for cancer diagnosis and follow-up has led to earlier detection of metastases, also in the oligometastatic setting, as defined by Hellman and Weichselbaum. Oligometastatic state has been defined as a state with 5 or fewer clinically detectable metastases, though no definitive consensus exists [3]. SBRT has shown prolonged progression-free survival (PFS) and overall survival (OS) rates in oligometastatic, oligoprogressive, and oligorecurrence settings from lungs, prostates, and others [4,5,6,7]. Oligometastases from gynecological cancers have been considered one of the most promising candidates for SBRT [8,9,10]. The incidence of isolated para-aortic lymph node (PALN) metastases after definitive treatment for carcinoma of the uterus has been reported to be between 1.7% and 12% [11,12]. Several retrospective series have reported pelvic exenteration and lymph node dissection as an option for selected patients with local recurrence [13]. SBRT is emerging as an attractive primary or alternative non-surgical salvage option for recurrent nodal pelvic and extra-pelvic tumors. First retrospective series of gynecological lymph nodes oligometastases treated with stereotactic technique were published as early as 2009, showing encouraging results [14]. In 2015, a review by Jereczek-Fossa et al. including 38 papers and 636 oligometastatic lymph nodes metastases treated with SBRT underlined the excellent in-field tumor control and safety of this treatment option [15]. Recent reviews and international guidelines already support radiotherapy as an option in the management of oligometastatic cervical cancer [16]. The aim of our study was to evaluate the efficacy and safety of SBRT as a therapeutic approach to lymph node recurrence in oligometastatic gynecological cancer patients.

## 2. Materials and Methods

### 2.1. Patients

Between November 2007 and October 2021, 22 patients (29 lesions) were treated with SBRT for gynecologic cancers with lymph nodes metastases. Most of the metastases originated from cervical cancer (14%), endometrial (65%), and ovarian cancer (21%). Oligo-recurrent, progressive patients were defined as patients with five or less new or enlarging metastases in an otherwise well-controlled disease status. Oligo-persistent disease was defined as five or less persistent lesions after systemic therapy. Before radiation therapy, patients were subjected to physical examination, blood chemistry test, total body computed tomography (CT) scan, though most patients did a whole body 18- F-fluorodeoxyglucose (FDG) positron emission tomography (PET-TC) scan. All cases were discussed at multidisciplinary meetings in the presence of medical oncologists. All patients gave written and informed consent before starting treatment.

### 2.2. Radiotherapy

Patients underwent CT simulation in a supine position with a slide of 2.5 mm. All patients were immobilized using a vacuum-assisted body mold to recreate exact positioning during daily sessions. Target lesion was not readily identified on the CT simulation, and the planning data set was registered to a diagnostic contrast CT or PET-CT, using a mutual information algorithm from our in-house treatment system, to facilitate gross tumor volume (GTV) delineation. A 3–8 mm isotropic expansion was generated from GTV to obtain planning target volume (PTV). Organs at risk (OARs) were delineated depending on the target lesion location without margins. The treatment planning system was Eclipse 4.5.5 (Varian), and VMAT/IMRT technique on a 6-MV linear accelerator Varian were used for treatment. The dose of SBRT was converted to the biologically effective dose (BED) to compare different dose-fractionation schedules. The BED was calculated using the linear-quadratic model with α/β = 10 Gy for the tumor and α/β = 3 to the organs at risk. Dose schedules were chosen with the aim of delivering ablative treatments respecting dose constraints for OARs.

### 2.3. Chemotherapy

A total of 19 (86%) patients received chemotherapy in addition to SBRT. Nineteen (86%), two (9%) and five (23%) patients received pre-SBRT, concurrent, and adjuvant chemotherapy, respectively.

### 2.4. Toxicities and Follow-Up

Tumor response was classified according to the RECIST criteria version 1.1 [17]. PET Response Criteria in Solid Tumors (PERCIST) were used to evaluate metabolic response in patients who underwent PET scans after SBRT [18]. Acute toxicities were reported according to the RTOG/EORTC scoring system, 5–6 weeks post-SBRT, and 3 months post-SBRT. Late toxicities were scored after a 6-month from SBRT according to the SOMA (symptoms, objective, management, analytic) scoring system [19,20]. CT scan with contrast and/or FDG/PET-CT were performed every three months for the first two years after treatment and every six months afterwards as follow-up to assess local failure or progression.

### 2.5. Statistical Analysis

Statistical analysis was performed using the SPSS statistical software package version 25.0 (SPSS, Chicago, IL, USA). OS was calculated from the date of diagnosis to the date of death from any cause or the date of the last follow-up. Local control (LC), distant metastasis free survival (DMFS) and PFS was calculated from the date of the end of radiotherapy course to the date of either distant metastases, locoregional recurrence or the date of the last follow-up. The Kaplan–Meier method was used to estimate the rates survival analysis. Univariate analysis for prognostic factors was performed with the log-rank test, and Cox proportional hazards regression was used to estimate hazard ratios (HR) to evaluate the association between factors and survival. *p* values less than 0.05 were deemed statistically significance.

## 3. Results

### 3.1. Patients and Treatment Characteristics

Patient, disease, and treatment characteristics are described in Table 1. Median age was 62 years (IQR, 50–80 years). Thirteen patients (60%) were <70 years and 9 patients (40%) were >70 years. LN metastases originated from endometrial carcinoma (65%), followed by ovarian cancer (21%) and cervical cancer (14%). LN location was 9 regional (31%) and 20 extra-regional (69%). The median time between the diagnosis of the primary tumor and the RT treatment was 33 months (IQR, 12–50.6 months). All patients received systemic therapy, 19 patients (86%) neoadjuvant, 2 (9%) concomitant, and 5 (23%) adjuvant at the time of oligoprogression.

The median diameter of the lesion was 1,9 mm (IQR: 1.5–2.6 mm). Treatments were prescribed to the median 98% isodose line (91–98%). The median GTV was 5.7 cc (IQR: 1.8–9.6 cc). The median PTV was 14.3 cc (IQR: 7.8–41.7 cc). The median SBRT dose was 36 Gy (IQR: 30–36 Gy), delivered in 1 to 8 fractions (median three fractions). In terms of BED10, the median delivered dose was 75.9 Gy (IQR: 58.5–79.2 Gy). Additional dosimetric and parameters of planning treatments are shown in Table 2.

### 3.2. Treatment Outcomes

**Figure 1:**
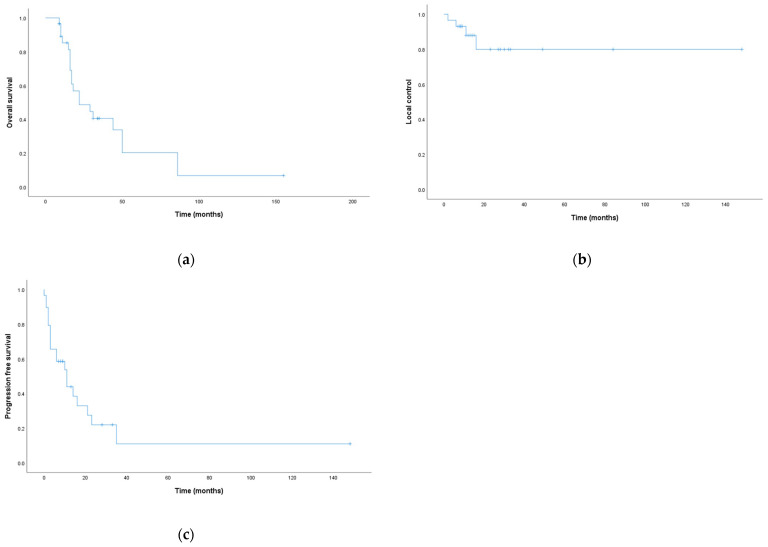
Kaplan–Meier curves for survival analysis: (**a**) overall survival; (**b**) local control; (**c**) progression-free survival.

With a median follow-up time of 17 months (IQR 10.5–31 months),the median survival was 22 months (CI 95%: 4.2–39.7, IQR: 12.5–34.5 months) with 6 months, 1 year, and 2 year OS at 96.6%, 85.2%, and 48.7%, respectively (Figure 1a). Median LC was not reached, and the 6 months, 1 year, and 2 year were 93.1%, 87.9%, and 79.9%, respectively (Figure 1b). Median PFS was 11 months (CI 95%: 4.28–17.7), and the 6 months, 1 year, and 2 year were 65.5%, 44%, and 22%, respectively (Figure 1c).

Median DMFS was 14 months (CI 95%: 7.7–20.2), and the 6 months, 1 year, and 2 year were 79.3%, 53%, and 37.1%, respectively. Clinical response after radiation therapy was evaluated using RECIST and PERCIST criteria and revealed a local overall response rate (ORR) of 90% (CR = 62%, PR = 28%), a stable disease in one patient (3%), and progression of disease in two patients (7%). Specific treatment outcomes are shown in Table 3.

No patients were subjected to re-irradiation, but the best supportive care was performed. The univariate analysis of prognostic factors for the OS, LC, and DMFS rates are shown in Table 4.

**Figure 2:**
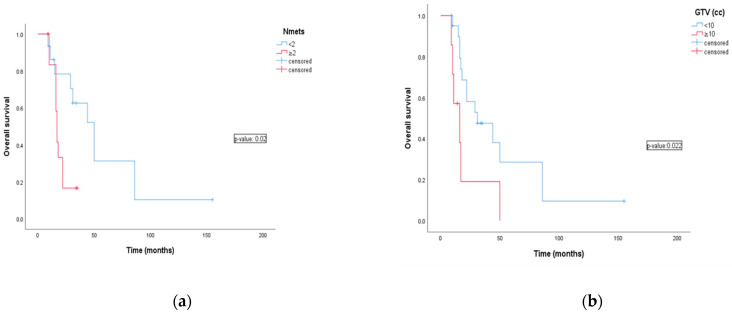

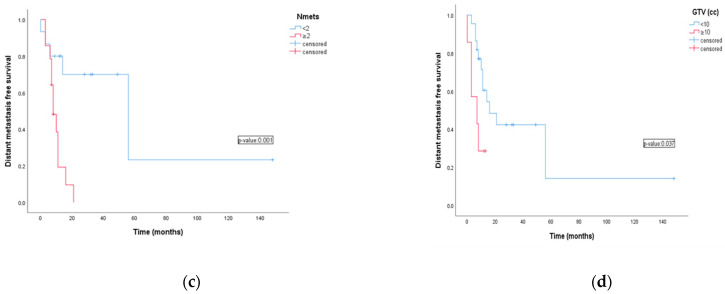
The effects of the potential prognostic factors on survival as analyzed by univariate analysis using log-rank test. (**a**) Overall survival for number of lymph nodes; (**b**) overall survival for Gross Tumor Volume (cc); (**c**) distant metastasis free survival for number of lymph nodes; (**d**) distant metastasis free survival for Gross Tumor Volume (cc).

The number of lymph nodes metastasis was a prognostic factor. One and two years OS were 86.2% and 78.3% for patients with <2 lymph nodes metastases compared to 83.3% and 33.3% for patients with >2 (HR: 3.32 CI 95%:1–10; *p*-value 0.022). Six month and one year DMFS were 80% and 70% vs. 78.6% and 38.6% for patients with <2 lymph nodes metastases and patients with >2. (HR: 5.57 CI 95%: 1.7–18; *p*-value 0.001). Lymph node metastases smaller then 10cc (GTV < 10 cc) and with diameter < 20 mm were significantly related with favorable outcomes of OS (*p*-value 0.022 and 0.024) and DMFS (*p*-value 0.037 and 0.045) compared to metastases >10 cc or with diameter >20 mm (Figure 2).

Better outcomes in terms of OS were observed based on the time between primary diagnosis and SBRT on lymph nodes metastasis. Patients treated >30 months vs. <30 months showed OS at 1 year and 2 year of 92.9% and 64.3% vs. 76% and 28.5%, respectively (HR: 3.09 CI 95%: 1.8–8; *p*-value 0.024). Although the dose in EQD2 > 60 Gy group had a significantly better OS than the dose in EQD2 < 60 Gy group (*p* = 0.05), there was no significant difference in DMFS between the groups. No difference regarding BED was found to be statistically significant among analyzed survival outcomes (OS, LC and DMFS). In contrast, LC did not show apparent correlation with any parameters. In terms of toxicities, four patients (18%) experienced acute G1-G2 toxicities. Of these patients, two developed G1 asthenia, one G2 asthenia, and finally one G1 nausea. In patients with asthenia, supplements chosen by our institute were prescribed, while only the patient with nausea was subjected to antiemetic drugs.

All acute symptoms were well treatable and were already decreasing after a short time. No G3–4 acute toxicity was reported, and no late toxicity was observed.

## 4. Discussion

Radiotherapy treatments for metastatic or recurrent gynecologic malignancies have historically been limited to supportive care and palliation with an unfavorable prognosis. Hellman and Weichselbaum theorized that for selected patients, there may be a state between localized and metastatic disease with a few sites of metastatic. Whereas there is no strict consensus about the number of metastases and/or sites, it is increasingly and widely accepted that oligometastatic disease represents the state of disease before tumor cells acquire aggressive widespread systemic metastatic potential [3]. Therefore, oligometastases could be amenable to local metastases directed treatments, such as surgical approach or ablative conformal radiation therapy.

Stereotactic Radiation for the Comprehensive Treatment of Oligometastases (SABR-COMET) phase II trial has recently published its long term results. Ninety-nine oligometastatic patients (1 to 5 metastases) were randomized to receive SBRT plus standard-of-care (SoC) systemic therapy versus SoC alone (2:1 ratio). At a median follow-up of 5.7 years, patients in SBRT arm had favorable 8 year OS (27.2% vs. 13.6%) and PFS (21.3% vs. 0.0%) compared to SoC arm patients. Approximately 20% of patients treated with SBRT lived beyond 5 years without recurrence or distant failure. Despite higher rates of toxicities in SBRT arm, globally SBRT was well tolerated and did not statistically impact on quality of life scores [7].

In our study, we employed SBRT rather than surgical treatment for oligo-persistent, oligo-recurrent, oligo-progressive metastases from gynecological primary malignancies.

Kunos et al. reported the results of a phase II clinical trial of robotic SBRT for metastatic gynecologic cancers. SBRT sites were PALNs in 19 (38%) patients, pelvic lymph nodes or soft tissue in 14 (28%) patients, and other sites in 17 (34%) patients. They reported that the radiation target response rate, including the complete response and partial response rates, was 96%, and that there was no treated clinical or radiological tumor relapse in SBRT sites. Among the whole cohort of patients, at final follow-up time analysis, 19 (38%) patients were free from disease. These data lead authors to cautiously conclude SBRT may concur to improve at least progression-free survival [21].

In 2016, Mendez et al. published a systematic review describing the emerging role of SBRT in different gynecological malignancies setting and analyzing available data on local control and treatment related acute and late toxicities. Among 375 gynecological cancer patients included in the analysis, more than half of the population received SBRT for pelvic or para-aortic lymph node metastases. Six studies and 183 patients were analyzed. At a median range of follow-up of 4–20 months, high rates of LC were reported (83%) with acceptable and safe toxicity profile (3.8% of grade 3–4 GI toxicities and only one grade 3 GU toxicity). Based on those findings, taking into account that no well-established and reliable option exists, authors suggest that current literature strongly supports SBRT as an alternative ablative treatment option for recurrent pelvic and para-aortic LN sites [22].

In the multi-institutional study of Ito et al. 113 patients, who had 1 to 5 abdominal/pelvic LN oligometastases, were treated with SBRT. Fifty-two (46%) patients primary sites were gynecological: 27 (24%) cervical cancer, 15 (13%) endometrial cancer and 10 (9%) ovarian cancer. The rate of 2-year OS, LC and PFS were 61.1%, 59.7% and 19.4%, respectively. They analyzed the outcomes in two subgroups according to EQD2 dose equivalent (<60 Gy and >60 Gy) and showed that the high-dose group did not significantly improve the two year OS on analysis of the entire cohort. Sixty-one (54%) LN treated were the only oligometastatic site and among this group, 40 (66%) LN were single PALN metastases. Isolated oligometastases subgroup receiving high-dose radiotherapy, as compared to multiple metastasis, showed statistically significant favorable 2-year OS (88.8% vs. 56.3%, *p*  =  0.009) and LC (88.8% vs. 56.3%, *p*  =  0.009). Notably, the high-dose group was composed mostly by cervical and endometrium primary sites. Those data emphasize once more, according to recent literature, that there could be a subgroup of patients more likely to benefit from SBRT treatments [23].

Mesko et al. retrospectively reported local response rates and PFS of 47 oligometastatic lesions from 28 gynecological cancer patients treated with SBRT. Among this cohort, 20 (42%) patients were treated on pelvic or PALN. Median SBRT dose for LN lesions was 37.5–40 Gy in 5 fractions. No local failure was reported for lymph-nodal targets treated. Only 6 (30%) patients had distant failure and none in the treated nodal chain. Smaller tumor diameters were significantly related to favorable local response (SD, PR and CR) as compared to lesions with unfavorable progressive disease (17.2 vs. 57.6 mm, *p* = 0.0044). Notably PALN and pelvic LN metastases analyzed had median tumor volume of 19 and 12 mm, respectively, which can support the very high local control rates (100%) in this subgroup. Higher prescription doses (BED > 79 Gy) were also related to better LC rates versus lower doses (BED < 59.6 Gy) [24].

The Italian multicenter retrospective study published by Macchia G. et al. analyzed the efficacy and safety of SBRT in patients with oligometastatic ovarian cancer. The median follow-up was 22 months and the 2-year actuarial LC rate was 81.9%. For patients aged ≤60 years, PTV ≤18 cm^3^, lymph node disease, and BED α/β10 > 70 Gy were associated with higher probability of CR in the multivariate analysis [25]. Similarly, in our study, the presence of <2 lymph nodes oligometastases was associated with significantly favorable OS at 1 year and 2 years compared with >2 (86.2% and 78.3% vs. 83.3% and 33.3%, *p* = 0.002). Additionally, in the univariate analysis the dose in EQD2 > 60, the Gy group had significantly better OS than the dose in EQD2 < 60 Gy group (*p* = 0.05).

Hasan et al. reported survival outcomes of SBRT for a cohort of 30 patients with recurrent gynecological cancer. The 5-year survival rate was 42% with a median survival period of 43.4 months. The median SBRT dose was 27.5 Gy (range 15–40 Gy) in 3 to 5 fractions. Multivariate analysis showed ECOG performance status (PS) and CTV to be independent prognostic factors for survival outcomes. The CTV was less than 24 cc yielded 2 year survival of 86 vs. 53% for greater than 24 cc (*p* = 0.005) [26]. In her study, B A. Jereczek-Fossa evaluated SBRT for single abdominal lymph node cancer recurrence in oligometastatic settings. Primary diagnoses included urological, gastrointestinal, gynecologic, and other malignancies. PFS was significantly correlated to the volume of target lesion; increase in GTV of 1 cm^3^ was correlated with 1% increase of the progression probability. Overall survival rate was significantly related to the primary histology [27]. Additionally, the study of Sato A. et al. analyzing SBRT to only lymph nodes from different primitive origin has showed that OS was significantly lower in a group of patients with a GTV > 20 cm^3^ compared with patients with smaller target lesion [28]. In our group of patients, GTV was prognostic factor for OS, the 1 and 2 year overall survival for GTV < 10 cc were 95.2% and 52.1%, respectively. Instead, the group for GTV > 10 cc the 1 and 2 year overall survival were 57.1% and 19.2% (*p*-value 0.022). GTV diameter was also an unfavorable risk factor for OS and DMFS.

Onal C et al. analyzed the clinical outcomes of SBRT in the treatment of patients with recurrent or oligometastatic ovarian or cervical tumor. The 1- and 2-year OS rates were 85% and 62%, respectively, and the 1 and 2 year PFS rates were 27% and 18%, respectively. Multivariate analysis showed that the early progression (≤12 months) and complete response were significant prognostic factors for improved survival [29]. In 2018 Iftode C et al. analyzed SBRT to 44 metastatic lesions in 26 patients (lymph nodes, 63.6%; liver, 31.8%; and lung, 4.5%) from ovarian cancer. The median LC was not reached. One-, 2-, and 5-year LC were 92.9%. The median PFS was 19 months, with 1 year PFS of 69.3%, 38% at 2 years, and 19% at 5 years. The median OS was 64.5 months, with all patients alive after 1 year, 92.7% at 2 years, and 61.7% at 5 years [30]. The present study, in line with the current literature, demonstrated excellent local control and safety of stereotactic body radiotherapy in the treatment of oligometastatic gynecological cancers. The actuarial 1 year and 2 year LC rates were 87.9% and 79.9%, respectively. The median OS time after completion of SBRT lesions was 22 months, and the actuarial 6 months and 1 year OS rates were 96.6% and 85.2%, respectively.

The current study is limited mainly by its retrospective nature, patient sample size, histology, and radiosensitivity heterogeneity of the analyzed cohort and different dose prescriptions.

## 5. Conclusions

SBRT in oligometastatic gynecological cancer is safe and feasible and provides good local control with a lower toxicity profile. The size of target lesion, the number of oligometastases and the time of radiation therapy seems to be significant prognostic factors. Further prospective studies are warranted to identify which subgroup of patients may benefit the most from this treatment.

## Figures and Tables

**Table 1:** Clinicopathological characteristics of the patients.

Characteristic	N (%) Median (IQR)
Age	62 (50–80)
<70 years	13 (60%)
>70 years	9 (40%)
ECOG	
0	18 (82%)
1	4 (18%)
Primary histology	
Ovarian Adenocarcinoma	5 (21%) 5
Cervical Adenocarcinoma Squamocellular	3 (14%) 2 1
Endometrial carcinoma Adenocarcinoma	14 (65%) 14
Lymph nodes metastases location	
Regional LN	9 (31%)
Extraregional LN	20 (69%)
Metastatic state	
Oligometastatic	8 (27%)
Olgorecurrent	14 (48%)
Oligoprogression	6 (21%)
Oligopersistent	1 (4%)
Timing of metastasis	
Synchronous	3 (10%)
Metachronous	26 (90%)
Chemotherapy	
Neoadjuvant	19 (86%)
Concurrent	2 (9%)
Oligoprogression	5 (23%)

ECOG: Eastern Cooperative Oncology Group status; LN: lymph nodes.

**Table 2:** Dosimetric characteristics.

Characteristic	No. (%) or Median (IQR)
SBRT treatment	
Total dose (Gy)	36 (30–36)
Fractions (n)	3 (1–8)
Dose per fraction (Gy)	10 (6–12)
BED (Gy)	75.9 (58.5–79.2)
EQD2 (Gy)	63.2 (48.7–66)
EQD2 < 60 Gy	12 (41%)
EQD2 > 60 Gy	17 (59%)
Isodose line (%)	98 (91–98)
GTV (cc)	5.7 (1.8–9.6)
PTV (cc)	14.3 (7.8–41.7)
Dm GTV (mm)	1.9 (1.5–2.6)
Planning technique	
VMAT	19 (66%)
IMRT	10 (34%)

BED: biologically effective dose; Dm: diameter; EQD2: equivalent 2 Gy dose; GTV: gross tumor volume; IMRT: intensity-modulated radiotherapy; PTV: planning tumor volume; SBRT: stereotactic body radiotherapy; VMAT: volumetric-modulated arc therapy.

**Table 3:** Treatment outcomes.

Outcome	Total (%) (IQR)
Follow-up	
Median (range)	18 (10.5–31)
Median survival	22 (12.5–34.5)
Radiological response	
CR	18 (62%)
PR	8 (28%)
SD	1 (3%)
PD	2 (7%)
Acute toxicities	
G1-G2	4 (18%)
No	18 (82%)

CR: complete response; G: grade; PD: progression disease; PR: partial response; SBRT: stereotactic body radiotherapy; SD: stable disease.

**Table 4:** Univariate analysis of survival by explicative variables.

		Overall Survival			Local Control			Distant Metastasis Free Survival		
Variable	Classifications	HR	95% CI	*p*-Value	HR	95% CI	*p*-Value	HR	95% CI	*p*-Value
Age, years	>70 vs. <70	1.78	0.7–4.5	0.22	0.55	0.05–5.4	0.61	0.86	0.3–2.4	0.77
Histologies	Endometrial vs. cervix vs. ovary	0.58	0.3–1	0.18	1.43	0.4–4.4	0.48	0.74	0.4–1.3	0.52
N of mets	≥2 vs. <2	3.32	1–10	**0.020**	5.4	0.5–57.1	0.12	5.57	1.7–18	**0.001**
Systemic therapy	Neoadjuvant vs. concurrent/adjuvant	0.46	0.1–1.3	0.13	1	0.09–10.5	0.98	0.73	0.2–2	0.53
Time primary-RT (months)	<30 vs. ≥30	3.09	1–8.8	**0.024**	0.19	0–80	0.08	2.48	0.8–6.8	0.066
BED10 (Gy)	<70 vs. ≥70	1.98	0.7–5.3	0.16	1.71	0.2–12.4	0.58	0.96	0.3–2.6	0.94
	<60 vs. ≥60	1.3	0.4–3.6	0.59	0.68	0.07–6.6	0.74	0.98	0.3–2.7	0.97
EQD2 (Gy)	<60 vs. ≥60	2.5	0.9–6.9	**0.050**	1.7	0.2–12.4	0.58	1.34	0.5–3.5	0.55
GTV (cc)	≥10 vs. <10	2.95	1.1–8	**0.022**	0.3	0.04–1.97	0.35	3.03	0.9–9.3	**0.037**
Dm GTV (mm)	≥20 vs. <20	2.93	1–7.9	**0.024**	0.44	0.04–4.3	0.47	2.71	0.9–7.6	**0.045**
PTV (cc)	≥15 vs. <15	1.65	0.6–4	0.23	0.41	0.04–4.3	0.48	1.61	0.6–4.1	0.28

BED: biologically effective dose; Dm: diameter; EQD2: equivalent 2Gy dose; GTV: gross tumor volume; HR: hazard ratio; mets: metastasis; N: number; PTV: planning target volume; RT: radiotherapy.

## Data Availability

Not applicable here.
